# A Comprehensive Analysis of Non-Desmosomal Rare Genetic Variants in Arrhythmogenic Cardiomyopathy: Integrating in Padua Cohort Literature-Derived Data

**DOI:** 10.3390/ijms25116267

**Published:** 2024-06-06

**Authors:** Maria Bueno Marinas, Marco Cason, Riccardo Bariani, Rudy Celeghin, Monica De Gaspari, Serena Pinci, Alberto Cipriani, Ilaria Rigato, Alessandro Zorzi, Stefania Rizzo, Gaetano Thiene, Martina Perazzolo Marra, Domenico Corrado, Cristina Basso, Barbara Bauce, Kalliopi Pilichou

**Affiliations:** Department of Cardio-Thoraco-Vascular Sciences and Public Health, University of Padua, 35121 Padua, Italy; maria.buenomarinas@unipd.it (M.B.M.); marco.cason@unipd.it (M.C.); riccardo.bariani@gmail.com (R.B.); rudy.celeghin@unipd.it (R.C.); monica.degaspari@unipd.it (M.D.G.); serena.pinci@studenti.unipd.it (S.P.); alberto.cipriani@unipd.it (A.C.); ilaria.rigato@unipd.it (I.R.); alessandro.zorzi@unipd.it (A.Z.); s.rizzo@unipd.it (S.R.); gaetano.thiene@unipd.it (G.T.); martina.perazzolomarra@unipd.it (M.P.M.); domenico.corrado@unipd.it (D.C.); barbara.bauce@unipd.it (B.B.); kalliopi.pilichou@unipd.it (K.P.)

**Keywords:** arrhythmogenic cardiomyopathy, dilated cardiomyopathy, “non-desmosomal” genes, *FLNC*, *TMEM43*, *DES*

## Abstract

Arrhythmogenic cardiomyopathy (ACM) is an inherited myocardial disease at risk of sudden death. Genetic testing impacts greatly in ACM diagnosis, but gene-disease associations have yet to be determined for the increasing number of genes included in clinical panels. Genetic variants evaluation was undertaken for the most relevant non-desmosomal disease genes. We retrospectively studied 320 unrelated Italian ACM patients, including 243 cases with predominant right-ventricular (ARVC) and 77 cases with predominant left-ventricular (ALVC) involvement, who did not carry pathogenic/likely pathogenic (P/LP) variants in desmosome-coding genes. The aim was to assess rare genetic variants in transmembrane protein 43 (*TMEM43*), desmin (*DES*), phospholamban (*PLN*), filamin c (*FLNC*), cadherin 2 (*CDH2*), and tight junction protein 1 (*TJP1*), based on current adjudication guidelines and reappraisal on reported literature data. Thirty-five rare genetic variants, including 23 (64%) P/LP, were identified in 39 patients (16/243 ARVC; 23/77 ALVC): 22 *FLNC*, 9 *DES*, 2 *TMEM43*, and 2 *CDH2*. No P/LP variants were found in *PLN* and *TJP1* genes. Gene-based burden analysis, including P/LP variants reported in literature, showed significant enrichment for *TMEM43* (3.79-fold), *DES* (10.31-fold), *PLN* (117.8-fold) and *FLNC* (107-fold). A non-desmosomal rare genetic variant is found in a minority of ARVC patients but in about one third of ALVC patients; as such, clinical decision-making should be driven by genes with robust evidence. More than two thirds of non-desmosomal P/LP variants occur in FLNC.

## 1. Introduction

Arrhythmogenic cardiomyopathy (ACM [MIM: 107970]) is an autosomal dominant inherited disorder of the myocardium with a heterogeneous clinical presentation including life-threatening arrhythmias at risk of sudden cardiac death (SCD), especially in the young and athletes [1,2].

ACM clinical diagnosis is multiparametric according to the 2010 Task Force Criteria (TFC) [3] and a pathogenic/likely pathogenic (P/LP) variant in an ACM-related gene is considered a major criterion. About 40% of ACM index cases carry one or more variants in genes encoding for proteins of the desmosomes (i.e., *plakophilin-2*, *PKP2*; *desmoplakin*, *DSP*; *desmoglein-2*, *DSG2*; *desmocollin-2*, *DSC2*; and *junctional plakoglobin*, *JUP*), which ensure the mechanical interaction of myocytes [4]. ACM displays reduced penetrance with intrafamilial expression variability [3,4,5]. Genetic architecture of ACM became more complex with the introduction of sequencing technologies, i.e., exome and genome sequencing, which led to the identification of numerous variants in cardiac disease-related or -unrelated non-desmosomal genes [6] such as *Transmembrane protein 43* (*TMEM43*) [7], *Desmin* (*DES*) [8], *Phospholamban* (*PLN*) [9], *Filamin C* (*FLNC*) [10], *Cadherin 2* (*CDH2*) [11,12] and *Tight junction protein 1 (TJP1)* [13]. Therefore, an incorrect attribution of a gene to ACM or classification of a genetic variant may lead to misdiagnosis.

Herein, we report the frequency of rare genetic variants in ACM-related non-desmosomal genes in a well characterized ACM cohort in order to understand their relevance in disease diagnosis and clinical management.

## 2. Results

**Figure 1:**
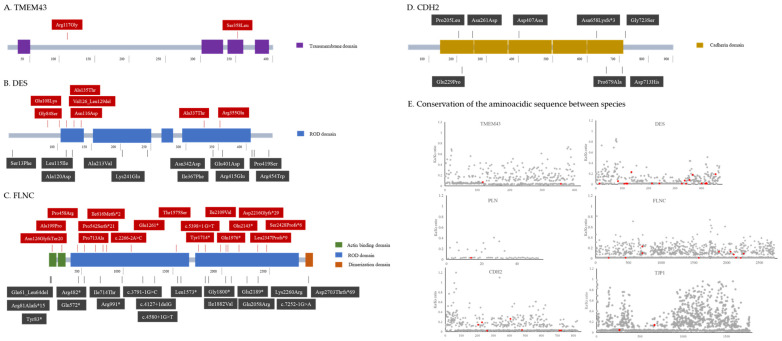
Schematic representation of protein structure and aminoacidic variants localization. (**A**) Transmembrane protein 43 (TMEM43). (**B**) Desmin (DES). (**C**) Filamin C (FLNC). (**D**) Cadherin 2 (CDH2). Variants found in our cohort are highlighted in red. (**E**) Plot with amino acid sequences running on the x-axis and the selective pressure represented by Ka/Ks ratio on the y-axis. Red dots represent amino acidic changes found in our cohort or reported in literature.

Our cohort comprised 320 ACM unrelated Italian index patients without desmosome-related P/LP variants: 243 (76%) with ARVC and 77 (24%) with ALVC. Overall, 35 rare genetic variants in non-desmosomal disease-related genes were identified in 39 index cases: 3 classified as P, 20 as LP, and 12 as variants of uncertain significance (VUS) (Table 1). Clinically, only 16 index cases had an ARVC phenotype, whereas 23 had a ALVC phenotype, counting 6.6% among the ARVC cohort (16/243) and 29.9% of ALVC (23/77), respectively (Table 1). No *PLN* and *TJP1* rare variants were identified in our cohort, but literature-reported variants were re-appraised (Figure 1, Table 2).

Twenty-three of the 165 studies obtained from the initial literature search satisfied eligible criteria for qualitative and quantitative analysis in ACM carriers of *TMEM43*, *DES*, *PLN*, *FLNC*, *CDH2*, and *TJP1* rare variants. In this setting, data collected from 104 index ACM cases highlighted 44 rare genetic variants in non-desmosomal disease-related genes (Table 2) of which 14 were classified as P, 19 as LP, 9 as VUSs, and 2 as benign.

### 2.1. TMEM43 Rare Variants and Cascade Genetic Screening

*Transmembrane protein 43* was linked to ACM in 2008 [7], with the founder c.1073C>T (Ser358Leu) variant as the only P variant reported in 22 patients/families out of 553 investigated index cases since then (Table 2). The median age to develop ACM related to Ser358Leu is 32 years (95% CI: 28–35) for males and 44 years (95% CI: 39–48) for females. The disease is fully penetrant in males by the age of 63 and in females by the age of 76 years.

In our cohort, c.1073C>T was identified in 5 unrelated patients (Figure S1—families A-B-C) (4 males, median age 42; 95% CI: 14–53) fulfilling TFC (Table 1). However, cascade genetic screening in these small pedigrees highlighted lower than 100% penetrance and high expression variability (Supplementary Results).

More recently, other *TMEM43* rare variants were reported [28,34] but without sufficient evidence to determine their pathogenicity. In this setting, we identified the VUS missense c.349A>G (exon 4, Arg117Gly) variant in a 22-year-old patient with definite TFC but without family history of ACM or SCD (Figure S1—family D, #1, II-1, male, see Supplementary Results).

### 2.2. DES Rare Variants and Cascade Genetic Screening

*Desmin* was linked to ACM in 2009 [8] with the founder variant c.38C>T (Ser13Phe) in index cases exhibiting variable phenotypes characterized by high-grade AV block, right-ventricle (RV) involvement and neurologic symptoms mainly affecting the lower limbs. The median age at presentation was 37 years for males (95% CI: 21–42) and 40.5 years for females (95% CI: 27–54). The disease is fully penetrant in males by the age of 49 and in females by the age of 51.5 years. To date, 14 rare variants have been reported in the literature in 21 ACM patients (Table 2) of which 6 classified as P, 6 as LP, 1 as VUS and 1 as benign.

**Figure 2:**
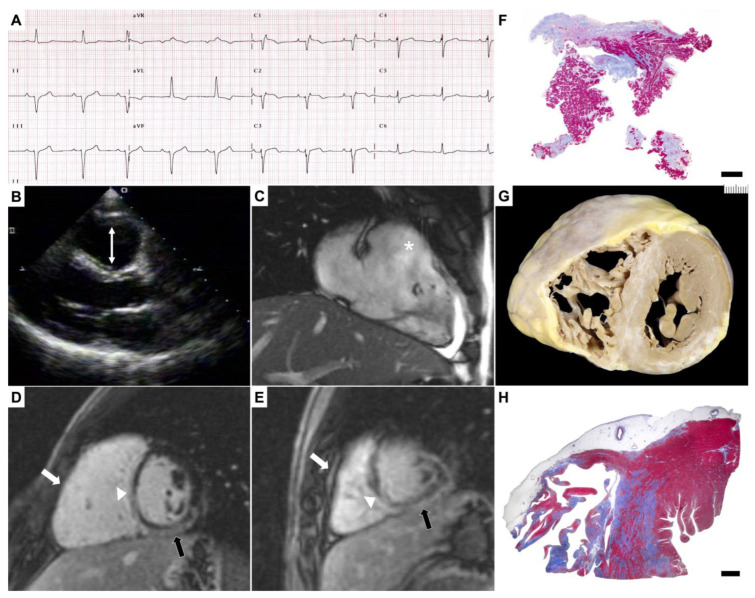
Clinical setting of 17-year-old *DES* carrier affected by left-dominant ACM. Clinical–pathological correlation in 17-year-old male (#9) affected by ACM with left-dominant pattern who underwent cardiac transplantation for refractory heart failure (missense variant c.346A>G carrier). (**A**) 12-leads ECG showing the typical epsilon waves in right precordial leads and T wave inversion on V1–V2. (**B**) Transthoracic echocardiogram with evidence of enlargement of right ventricular outflow tract (white arrow). (**C**) Cine-CMR confirming the dilatation (white asterisk). (**D**,**E**) Short-axis CE-CMR with LGE detectable on the entire RV wall (white arrow) and on the right side of the interventricular septum (white arrowhead). LV involvement in the inferior wall is also detectable (black arrow). (**F**) RV EMB demonstrating areas of replacement-type fibrosis and endocardial thickening (Heidenhain trichrome stain, panoramic view, scale bar 200 µm). (**G**) Gross view of the explanted heart with biventricular scars corresponding at histology (**H**) to transmural fibrosis of the RV free wall, subepicardial fibrosis in the LV and septal involvement on the RV-side (Heidenhaim trichrome stain, panoramic view, scale bar 5 mm).

In our cohort, genetic screening in DES revealed 9 rare genetic variants in 9 different probands (6 males, median age 35; 95% CI: 17–65), 5 of which were located at exon 1 encoding for the head ROD 1A protein domain (Table 1, Figure 1). Altogether, the sequence variant spectrum of *DES* includes 89% missense variants (8 out of 9; exemplified case in Figure 2).

Overall, 6 of 9 rare DES variants identified in our cohort were classified as LP due to variant properties, specifically mutational hotspots according to critical and well-stablished protein domains (“Head” and “IF rod”) [35], literature evidence, and co-segregation analysis when available (Supplementary Results). Of note, the ALVC phenotype, both pathologically and clinically, is predominant in *DES* carriers, with a total of 6 index cases and 2 family members counting 66.7% of *DES* carriers. In detail, 55.6% of variants localize in the ROD 1A domain and comprise 3 cases with fatal event (one heart transplantation and two SCD).

### 2.3. FLNC Rare Variants and Cascade Genetic Screening

*Filamin c* was associated with ACM in 2016 [10], reporting 6 *FLNC* truncating variants in 7 patients (median age 38; 95% CI: 17–55) displaying infero-lateral negative T waves, mild to moderate LV systolic dysfunction, and regional dyskinesia. To date, 21 variants have been reported in the literature in approximately 53 ACM carriers, both index and family members (Table 2).

In our cohort, 22 *FLNC* rare variants (Table 1) were identified in 22 index cases (15 males, median age 45; 95% CI: 35–61) of whom 16 carried ‘radical’ variants (8 deletions/insertions, 6 nonsense and 2 splice site variants) classified as P/LP, and 6 carried missense variants classified as VUS. ALVC phenotype was diagnosed in 63.6% of index cases (n = 14). Cascade genetic screening was feasible in 5 of the 22 families (Figure S3), and 12 (7 males, median age 37; 95% CI: 32–63) of the 20 family members screened were *FLNC* carriers (8 radical, 4 missense—Supplementary Results).

### 2.4. CDH2 Rare Variants and Cascade Genetic Screening

In 2017, 2 P/LP *CDH2* genetic variants in 3 ACM patients were reported simultaneously by two independent studies [11,12] engaging a total cohort of 78 patients. A more recent multicentric effort on 500 unrelated gene-elusive ACM patients, added 6 more rare *CDH2* variants of which 4 were classified as P/LP (Table 2) [33]. Overall, 8 ACM index patients harboring 7 P/LP *CDH2* variants were described in the literature (4 males, median age 23, 95% CI: 14–59). However, disease penetrance and phenotypic characterization is still pending.

Among our 320 unrelated ACM patients only 2 *CDH2* carried variants of unknown significance (Table 1 and Figure S4).

### 2.5. Burden of Rare Genetic Variants in Non-Desmosomal Genes

Taken together data obtained from our cohort and the literature, a 3.79-fold enrichment of rare non-synonymous *TMEM43* variants was observed in ACM patients compared to the general population, with a frequency ranging from 1.9% (6/320) to 3.21% (28/873). Likewise, *DES* missense variants were enriched 10.31 times in ACM population and the frequency of this gene ranges from 2.8% (9/320) to 6.2% (30/486). *FLNC* rare non-synonymous variants frequency was calculated about 6.9–5.1% (22/320-42/818) but significant enrichment (107-fold) in the ACM population was evident only when considering radical variants (31/818).

Only 2 *CDH2* VUS were found in our cohort (0.62%) summed with the scant literature evidence (10/898, 1.1%), leading to *CDH2* genetic variant frequency estimation as 1%. This remark was further supported by no significant differences at the burden testing between ACM and control population for *CDH2* rare non-synonymous variants.

As no rare *PLN* or *TJP1* variants were found in our cohort, frequency calculation was based only upon literature data. Approximately 4.6% (31/670) of the affected ACM population may carry rare genetic variants in *PLN*, greatly influenced by the founder effect of the unique radical variant in the Dutch population. In *TJP1*, frequency was estimated about 0.55% (2/360) and no enrichment of rare genetic variants was evident probably due to the small cohort size evaluated. On the contrary, *PLN* was enriched over 100 times in ACM population due to its characteristic founder effect (Table 3).

### 2.6. Missense Variants Occur at Sites under Negative Selection

Evolutionary conserved sequences within proteins among species are crucial to maintain protein function and structure. In evolution, selection is a powerful force which works by constantly sorting through the variation that is produced by mutations to select the fit and remove the unfit, while ignoring neutral changes. As such, protein sequences of target genes were analyzed by studying the conservation of amino acids among 10 species calculating the Ka/Ks ratio, meaning the rate of non-synonymous substitutions to the rate of synonymous substitutions (Figure 1). TMEM43, DES, PLN and FLNC, are highly conserved protein sequences, with Ka/Ks ratio near 0, indicating negative selection pressure which tends to remove harmful variants. Instead, CDH2 presents a positive selection pressure particularly in 3 amino acids (Ka/Ks ratio > 1), indicating that missense variants in CDH2 tend to be enriched improving the fitness and consequently have less impact in protein structure. Although TJP1 is also under negative pressure, it is possible to observe how most amino acids have a ratio over 0.8, especially in the last 700 amino acids, indicating a less conservative region.

However, all missense variants examined in this study, obtained both from our cohort and the literature, are located in residues under negative selection pressure with Ka/Ks ratio < 0.3 as such were further assessed (Figure 1).

## 3. Discussion

About half of ACM patients fulfilling classical TFC harbor a causative rare variant in genes encoding cardiac desmosomal proteins [4]. Many non-desmosomal genes have been recently linked to ACM pathogenesis displaying low disease penetrance and anecdotal frequencies in disease cohorts [36]. In the clinical setting, current variant adjudication guidance is important for translating genetic data into information relevant to clinical diagnosis and family care. However, clinical genetic testing panels feature many disputed or limited-evidence genes. The present study provides a rigorous evaluation of rare variants in *TMEM43*, *DES*, *PLN*, *FLNC*, *CDH2*, and *TJP1* genes and their incidence in ACM [7,8,9,10,11,12,13], integrating recent efforts from international scientific and clinical panels [6,37] to define the genetic architecture of monogenic ACM and its implication in the clinical management. Indeed, *PLN* and *TMEM43* contribute to disease pathogenesis, particularly in geographic regions with well-characterized founder variants [7,9]. *DES* was moderately associated with ACM by the ClinGen framework [6], whereas *CDH2* and *TJP1* belong to the category of genes recently associated with the disease with limited evidence of one or few rare P/LP variants detected in small families with ACM [6]. For these genes, we replicated the screening in large cohort of gene elusive ACM cases in order to upgrade or not, the level of evidence.

### 3.1. Disputed Genes

The genes *TJP1* and *CDH2* exhibited a weak gene–disease relationship:

*Tight Junction Protein 1* gene, which encodes the multifunctional protein ZO-1 interacting with different proteins of cell–cell junctions in the so-called area composita, was linked to the disease phenotype in 2018 [38]. Specifically, two rare variants were annotated in a mixed cohort of 40 Italian–Dutch–German ACM patients [13]. Diseases associated with *TJP1* include neovascular glaucoma and brain oedema and *TJP1* related pathways include G-Beta gamma signalling and E-cadherin signalling in the nascent adherens junction. However, the absence of rare variants in the *TJP1* gene in our local cohort of 320 ACM patients is well matched to the non-significant burden test obtained from literature data; as to support that this exceptional finding needs larger disease cohorts and more experimental evidence in order to assess its role in the disease pathogenesis.

The *Cadherin-2* gene encodes a Ca^2+^-dependent cell adhesion protein, previously known also as N-cadherin, with a vital role in the intercalated disc [38]. Diseases associated with *CDH2* include agenesis of corpus callosum, cardiac, ocular and genital syndrome. In 2017, two independent studies demonstrated the implication of *CDH2* missense variants to ACM pathogenesis [11,12]. In our cohort we found two additional rare *CDH2* variants both classified as VUS. Overall, gene prevalence was estimated less than 1% and enrichment analysis of rare genetic variants failed to show any difference of their burden in ACM with respect the control population. This observation along with the positive selection pressure in *CDH2* reflect a disputed role of this gene in ACM pathogenesis.

### 3.2. Endemic Genes

The genes *PLN* and *TMEM43* exhibited strong gene–disease association in limited geographic regions based on the accumulation of convincing genetic and experimental evidences.

*Phospholamban* is a Ca^2+^-ATPase, which triggered the hypothesis that Ca^2+^ homeostasis might play an important role in the pathogenesis of arrhythmogenic phenotype in p.Arg14del carriers with ACM or dilated cardiomyopathy (DCM) [9]. Multiple studies [9,28,34,39,40] support the conclusion that this variant is a founder mutation diffuse in the Dutch population. Even though the burden test for *PLN* rare genetic variants in the ACM population exhibited a 100-fold enrichment, no P variants were found in our cohort of 320 Italian index cases. The absence of *PLN* genetic variants in our cohort might be due to the effect of geographic limits underlying the importance of cohort location and ethnicity in order to avoid misinterpretation of genetic variants. However, this does not explain the spectrum of clinical features in patients with this variant adequately to suggest the possible involvement of other players in the disease development [41].

*Transmembrane Protein 43* encodes for the formerly called LUMA protein, a four-transmembrane domain protein which interacts with lamins and emerins and is involved in the structural organization of the nuclear membrane [42,43]. Diseases associated with *TMEM43* include Emery–Dreifuss muscular dystrophy type 7, whereas other studies have linked *TMEM43* p.Ser358Leu variant to ACM as the founder pathogenic variant in limited regional cohorts [7,19,20,21]. Although this Ser358Leu is the only *TMEM43* variant with pathogenic evidence till now, in our cohort *TMEM43* frequency is about 1.9%. This frequency is similar to the one reported for some desmosomal genes, i.e., *DSC2* and *JUP* [4] and a 3.79-fold enrichment was annotated for this gene in ACM patients. In particular, 5 patients, 3 with ARVC and 2 with ALVC, were carriers of the already reported p.Ser358Leu while the no-previously described p.Arg117Gly was identified in the sixth proband. However, the four small pedigrees available exhibited a disease penetrance of less than 100%, often with no signs or symptoms in elderly family carriers, suggesting the need for additional genetic or epigenetic factors to develop ACM instead of DCM. Larger multicentric cohort studies should be undertaken to confirm its univocal role in ACM.

### 3.3. Definitive Gene–Disease Association

*Desmin* is a structural intermediate filament protein present in the cytoskeleton of the leiomyocytes, rhabdomyocytes, and cardiomyocytes, and it is associated with different cellular structures. Its function is related to the maintenance of the structural integrity of the cardiomyocyte [44]. DES-related myofibrillar myopathy is caused by missense rare genetic variants located in the central 2B domain of the protein, leading to skeletal muscle disease, which typically precedes cardiac involvement. However, P *DES* variants have also been described in DCM patients without skeletal muscle disease [45] and, in 2009, were linked to ACM typically affecting the RV or causing severe biventricular involvement [8,22]. However, an ALVC pattern was highlighted in our cohort, counted in 66.7% of *DES* carriers. Of note, fatal events (heart transplantation and SCD) at a young age were noticed in 33.3% of our index cases, in keeping with a predominant LV involvement and high risk of ventricular arrhythmias, reported recently by a multicentric study [46]. In contrast to this last [46], *DES* prevalence, combining the Padua and literature cohorts’ data, is more frequent (6.2%, 30/486) with an enrichment of 10.31 times in the overall ACM cohort. This finding supports a definitive association of *DES* to the disease. Although the exact mechanism by which a single amino acid change could lead to disease development needs to be better elucidated, it is evident the involvement of *DES* variants in ACM with ARVC or ALVC or DCM or left-ventricular non-compaction [47].

*Filamin C* encodes gamma filamin, which is a muscle-specific protein playing a central role in muscle cells, participates in the anchoring of membrane proteins to the actin cytoskeleton in response to signalling events and displays structural functions at the Z lines in muscle cells [48]. The frequency obtained from both Padua and the combined cohorts was in the range of 5.1%, confirming previously reported data on *FLNC*-related carriers (ranging from 3% to 7.5%) affected either by ACM or DCM [30,32]. However, this gene has not yet been adjudicated by recent efforts of the ClinGen evidence-based framework [6]. In this setting, burden testing of rare *FLNC* variants showed an astonishing 107-fold enrichment of radical variants in the ACM cohort, whereas this approach failed to show enrichment in the ACM population when radical and missense variants were summed together. Radical *FLNC* variants, which are expected to invoke haploinsufficiency via non-mediated decay, were found throughout the whole gene strongly associated to ALVC. This fact potentially could lead to Z-disc disarray and weakened cell–cell adhesion with subsequent impaired mechanotransduction. These structural alterations may promote fibrogenesis, contributing to the arrhythmogenesis, but functional data are still needed to determine the role of these variants in the ACM pathogenesis. Further, it is interesting to note that *FLNC* carriers demonstrated a later disease onset of the disease compared to desmosomal carriers, and muscular disabilities were not detectable or searched for. Specifically, only 5 of 34 *FLNC* carriers identified in our cohort fulfilled 2010 TFC showing a huge gap of these criteria in identifying ALVC which dropped disease incidence to 16.7%. Instead, 2020 Padua Criteria for ACM [49,50] were able to distinguish 8 ARVC, and 14 ALVC patients among *FLNC* carriers, estimating the disease penetrance about 71%.

### 3.4. Study Limitations

The small number of rare genetic variants in the most relevant non-desmosomal disease genes is well matched with the frequency reported in literature but does not exclude potential study bias. Co-segregation studies were limited due to the small number of relatives available for screening. The Padua cohort, and likely many cohorts derived from the literature, are gene-elusive ACM cohorts; as such the burden test analysis is referred to gene elusive ACM population. Public databases such as gnomAD provide some ancestry information; the exact ancestry of the individuals is not available and hence cannot be readily and directly compared to the ancestry of the case subjects. Potential bias derived from the use of gnomAD control database as control population cannot be excluded.

## 4. Materials and Methods

### 4.1. Literature Search and Study Selection

**Figure 3:**
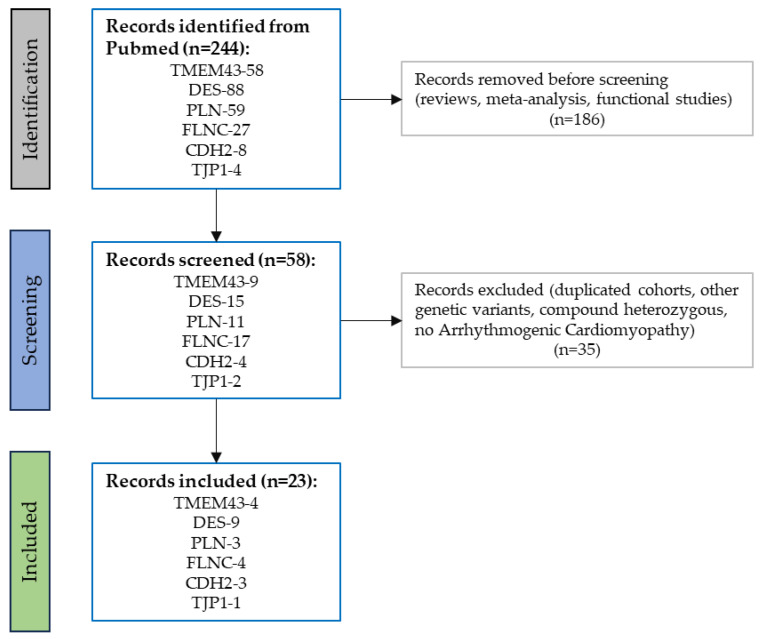
Flow diagram summarizing the literature review and inclusion/exclusion process.

Electronic search engines included PubMed/Medline and Scopus for the following search keyword string: TMEM43 OR DES OR PLN OR FLNC OR CDH2 OR TJP1 AND ACM (including all possible acronyms) were interrogated till August 2023. We also carefully reviewed reference lists of original publications and review articles for missing studies. Duplicates were eliminated (Figure 3).

These studies were filtered independently by three reviewers (M.C., M.B.M. and R.C.) and occasional disagreements that were unable to be resolved by the reviewers were settled by two additional authors (K.P. and C.B.). Only the first study published or the one containing more clinical details was included when the same cohort was reported by multiple studies fulfilling the inclusion criteria.

For each study, the following data were recorded: the first author, publication year, number of probands and family members tested, type of genetic analysis and genetic/aminoacidic variants.

### 4.2. Padua Cohort

The study cohort comprised 320 unrelated Italian index patients (233 males; median age: 45, 95% CI: 43–47) referred to the Referential Clinical Center for Inherited Cardiomyopathies of Padua. Specifically, 282 patients had a clinical diagnosis of ACM based on the 2010 Task Force Criteria (TFC) [3] or 2020 Padua criteria [49,50], in order to collect both dominant right-ventricular ACM with or without left-ventricular involvement (ARVC) and left-dominant ACM (ALVC); 38 index cases had a post-mortem diagnosis of ACM.

Prior genetic screening for single nucleotide variants or copy number variations in desmosome-encoding genes (*PKP2*, *DSP*, *DSG2*, *DSC2* and *JUP*) showed no P or LP variants based on current American College of Medical Genetics and Genomics (ACMG) recommendations [51,52].

Clinical evaluation consisted of a detailed personal and family history, physical examination, 12-lead ECG, 2-dimensional echocardiogram, signal-averaged ECG, and stress test ECG. CE-CMR was performed in selected cases with a previously detailed protocol [53].

The study was conducted according to the guidelines of the Declaration of Helsinki and approved by the Ethics Committee of Azienda Ospedale-Università Padova (protocol code 4501/AO/18 and date of approval 26 April 2018). Written informed consent was obtained from all subjects involved in the study.

### 4.3. Statistical Analysis

Statistical analysis was conducted with GraphPad Prism version 7.0 for Windows (GraphPad Software, La Jolla, CA, USA, www.graphpad.com). Age was expressed in terms of median ± 95%CI. Differences in variant distribution were assessed by Fisher’s exact test for contingency analysis. Burden test in terms of differences in rare variant weight between ACM and gnomAD control population (considering as controls) were calculated by Fisher’s exact test as previously reported [54]. ACM allele count for each gene was determined taking together gene-elusive ACM population from the selected literature and Padua cohort whereas control alleles were extrapolated from gnomAD control database. Enrichment range of rare variants for each gene was calculated by Woolf logit interval for odd ratio as “relative risk” of having a causative rare variant in a determine gene when belonging to ACM cohort compared to the risk when belonging to control population. For a Detailed Description see Supplementary Materials.

## 5. Conclusions

Clinical diagnosis of ACM is still challenging, and genetic testing is often considered decisive, particularly when dealing with early ARVC and ALVC. Our study demonstrates that non-desmosomal genes often lack of evidence of gene-disease association and should not drive clinical decision-making. Additionally, some genes should be taken into account only in specific cohort location and ethnicity to reduce VUS, but penetrance calculation should be systematically reappraised. High frequency of missense *DES* and radical *FLNC* variants is observed in patients with a ALVC phenotype and high disease penetrance, supporting their strong pathogenic role. Indeed, only 6.6% of ARVC patients carry a rare genetic variant in non-desmosomal genes, at difference from 29.9% of ALVC patients. Functional studies are mandatory to clarify the role of non-desmosomal rare genetic variants in ACM pathogenesis and multicenter studies will profit in clarifying population divergences underlying phenotypic variability.

## Figures and Tables

**Table 1:** Clinical data of 39 Caucasian carriers of non-desmosomal gene variants.

Pt#	Sex	Age (y)	Gene	HGVSc	HGVSp	ACMG	Previous Reference	ECG Low QRS Voltages	ECG TWI V1–V3	ECG TWI Infero-Lateral/ Lateral Leads	Major VA/ 24-h PVB Count	Echo Changes	CMR LVEF (%)	CMR LV Dilation	CMR RVEF (%)	CMR RV Dilation	CMR LV LGE	ACM Variant
#1	M	21	*TMEM43*	c.349A>G	p.Arg117Gly	VUS	Novel	-	+	-	nsVT	+	56	+	52	+	+	ARVC
#2	F	45	*TMEM43*	c.1073C>T	p.Ser358Leu	P	[7]	N/A	N/A	N/A	+	+	48	-	66	-	+	ARVC
#3	M	51	*TMEM43*	c.1073C>T	p.Ser358Leu	P	[7]	-	+	-	sVT	+	47	+	41	+	+	ARVC
#4	M	40	*TMEM43*	c.1073C>T	p.Ser358Leu	P	[7]	+	-	-	nsVT	+	49	+	48	+	-	ARVC
#5	M	53	*TMEM43*	c.1073C>T	p.Ser358Leu	P	[7]	+	-	-	sVT	+	N/A	N/A	N/A	N/A	N/A	ALVC
#6	M	14	*TMEM43*	c.1073C>T	p.Ser358Leu	P	[7]	N/A	N/A	N/A	sVT	N/A	57	+	N/A	N/A	+	ALVC
#7	F	39	*DES*	c.250G>A	p.Gly84Ser	VUS	Novel	-	-	-	N/A	+	58	-	N	-	+	ALVC
#8	M	35	*DES*	c.322G>A	p.Glu108Lys	LP	[14]	+	-	-	1415	+	58	-	64	-	+	ALVC
#9	M	17	*DES*	c.346A>G	p.Asn116Asp	LP	[15]	+	+	+	SCD	+	47	N/A	25	N/A	N/A	ARVC
#10	M	81	*DES*	c.403G>A	p.Ala135Thr	VUS	Novel	-	-	+	nsVT	+	37	+	56	-	+	ALVC
#11	M	29	*DES*	c.1009G>A	p.Ala337Thr	LP	Novel	N/A	N/A	N/A	SCD	N/A	N/A	N/A	N/A	N/A	N/A	ALVC
#12	M	58	*DES*	c.1064G>A	p.Arg355Gln	LP	[16]	-	-	-	250	+	62	-	55	-	-	ARVC
#13	F	30	*DES*	c.377_388delTGCGCTTCCTGG	Val126_Leu129del	LP	Novel	N/A	N/A	N/A	SCD	+	40	+	N/A	N/A	+	ALVC
#14	M	17	*DES*	c.833G>A	p.Arg278Gln	VUS	Novel	-	-	-	276	-	58	-	60	-	+	ALVC
#15	F	65	*DES*	c.407T>A	p.Leu136His	LP	[17]	+	+	+	11065	+	53	-	23	+	+	ARVC
#16	M	65	*FLNC*	c.1373C>G	p.Pro458Arg	VUS	[18]	+	-	-	sVT	+	70	-	43	-	-	ARVC
#17	M	40	*FLNC*	c.6325 A>G	p.Ile2109Val	VUS	[18]	-	+	-	4439	+	60	-	16	+	-	ARVC
#18	F	43	*FLNC*	c.4724C>G	p.Thr1575Ser	VUS	[18]	-	+	+	aSCD	+	66	+	33	-	+	AL VC
#19	M	56	*FLNC*	c.2137C>G	p.Pro713Ala	VUS	[18]	+	+	-	sVT	+	55	-	37	+	-	ARVC
#20	M	64	*FLNC*	c.595G>C	p.Ala199Pro	VUS	[18]	+	+	+	sVT	+	44	+	35	+	+	ARVC
#21	F	30	*FLNC*	c.5142C>G	p.Tyr1714*	LP	Novel	N/A	N/A	N/A	nsVT	+	51	+	N	-	+	ALVC
#22	M	17	*FLNC*	c.5926C>T	p.Gln1976*	P	[18]	N/A	N/A	N/A	SCD	N/A	N/A	N/A	N/A	N/A	N/A	ALVC
#23	M	43	*FLNC*	c.3781G>T	p.Glu1261*	LP	[18]	+	-	-	nsVT	-	58	-	61	-	+	ALVC
#24	F	66	*FLNC*	c.1623_1624insT	p.Pro542Serfs*21	LP	[18]	-	+	+	Rare	+	66	-	45	-	-	ARVC
#25	M	57	*FLNC*	c.1848_1852del	p.Ile616Metfs*2	LP	[18]	+	-	-	nsVT	+	46	-	49	-	+	ALVC
#26	M	63	*FLNC*	c.5398+1 G>T	/	LP	[18]	+	-	+	sVT	+	45	-	N	-	N/A	ALVC
#27	F	35	*FLNC*	c.7037dup	p.Leu2347Profs*9	LP	[18]	-	-	+	nsVT	+	39	+	56	-	+	ALVC
#28	F	42	*FLNC*	c.376_392del	p.Asn126Glyfs*20	LP	[18]	+	-	+	nsVT	+	34	+	44	-	+	ALVC
#29	M	49	*FLNC*	c.2266-2A>C	/	LP	Novel	-	-	+	sVT	+	65	+	N	N	+	ALVC
#30	F	33	*FLNC*	c.7282delT	p.Ser2428Profs*6	LP	Novel	-	+	-	N/A	+	N/A	+	NA	-	+	ALVC
#31	M	62	*FLNC*	c.6646dupG	p.Asp2216Glyfs*29	LP	Novel	-	-	+	sVT	+	44	+	50	-	+	ALVC
#32	M	42	*FLNC*	c.6427C>T	p.Gln2143*	LP	Novel	+	-	-	-	-	64	-	67	-	+	ALVC
#33	M	18	*FLNC*	c.560dup	p.Asp187Glufs*52	LP	Novel	-	-	-	1498	-	54	-	55	-	+	ARVC
#34	M	21	*FLNC*	c.4060C>T	p.Arg1354*	P	[10]	+	-	+	418	+	42	+	56	-	+	ALVC
#35	F	61	*FLNC*	c.1948C>T	p.Arg650*	LP	[10]	+	-	+	2730	+	37	+	57	-	+	ALVC
#36	M	47	*FLNC*	c.3009_3010insGCGGGTG	p.Thr1004Alafs*60	LP	Novel	+	-	-	9721	+	56	-	44	-	-	ARVC
#37	M	59	*FLNC*	c.2404G>A	p.Gly802Ser	VUS	Novel	-	-	-	5906	+	56	-	44	-	-	ARVC
#38	F	32	*CDH2*	c.2349+4 A>G	/	VUS	Novel	-	+	-	-	+	26	+	57	-	+	ARVC
#39	M	38	*CDH2*	c.*92 T>A	/	VUS	Novel	-	-	-	sVT	-	58	+	56	-	-	ALVC

+ positive/present; - negative/absent; ACM: arrhythmogenic cardiomyopathy; ACMG: American College of Medical Genetics and Genomics; ALVC: arrhythmogenic cardiomyopathy with prevalent left ventricular involvement; ARVC: arrhythmogenic cardiomyopathy with prevalent right-ventricular involvement (with or without left-ventricular involvement); aSCD: aborted sudden cardiac death; CDH2: Cadherin-2; CMR: cardiac magnetic resonance; DES: desmin; ECG: electrocardiogram; echo: echocardiogram; F: female; FLNC: filamin C; HGVS: Human Genome Variation Society; M: male; LD: left dominant; LGE: late gadolinium enhancement; LP: likely pathogenic; LV: left ventricle; LVEF: left-ventricular ejection fraction; N: normal; N/A: not available; nsVT: nonsustained ventricular tachycardia; P: pathogenic; PVB: premature ventricular beat; RV: right ventricle; RVEF: right-ventricular ejection fraction; SCD: sudden cardiac death; sVT: sustained ventricular tachycardia; TMEM43: transmembrane protein 43; TWI: T-wave inversion; VA: ventricular arrhythmias; VUS: variant of uncertain significance.

**Table 2:** Rare genetic variants in TMEM43, DES, PLN FLNC, CDH2, and TJP1 reported in literature.

Gene	HGVSc	HGVSp	Mutation Carrier			Probands Tested	Type of Analysis	ACMG	References
			Probands N.	Family Members Affected N.	Family Members Not Affected N.				
*TMEM43*	c.1073C>T	Ser358Leu	15	83	61	150	Haplotype + Sanger	P	Merner et al., 2008 [7]
			1	1	1	55 definite + 10 borderline	Sanger		Christensen et al., 2011 [19]
			6	1		195	Sanger		Baskin et al., 2013 [20]
	/	/	0			143	Sanger		Haywood et al., 2013 [21]
*DES*	c.38C>T	p.Ser13Phe	5	22		NA	Sanger sequencing	P	van Tintelen et al., 2009 [8]
	c.1024A>G	p.Asn342Asp	1	1		52	DGGE and Sanger sequencing for 5 ACM major genes	P	Otten et al., 2010 [22]
	c.1360C>T	p.Arg454Trp	1	1				P	
	c.347A>G	p.Asn116Ser	1	0	0	23		P	Klauke et al., 2010 [15]
	c.1255C>T	p.Pro419Ser	1	7			WES + sanger sequencing	P	Hedberg et al., 2012 [23]
	c.721A>G	p.Lys241Glu	1	0	0	91	WES + sanger sequencing	LP	Lorenzon et al., 2013 [24]
	c.638C>T	p.Ala213Val	1	0	0			B	
	c.359C>A	p.Ala120Asp	1	2		NA	Sanger sequencing	LP	Brodehl et al., 2013 [15]
	c.977A>G	p.His326Arg	1	1		NA		VUS	
	NA	p.Ile367Phe	2	9		NA	Sanger sequencing	LP	Rippol-vera et al., 2015 [25]
	NA	p.Pro419Ser	1	1		NA		P	
	NA	p.Arg415Glu	1	4		NA		LP	
	c.1203G>C	p.Glu401Asp	1	31	0	NA	NGS Target sequencing + Sanger sequencing	LP	Bermúdez et al., 2018 [26]
	c.343C>A	p.Leu115Ile	3	7	0	138	WES	LP	Protonotarios et al., 2020 [27]
*PLN*	c.40_42del	p.Arg14del	12	NA	NA	97	Sanger sequencing	P	van der Zwaag et al., 2012 [9]
			19	NA	NA	142	Sanger sequencing		Groeneweg et al., 2013 [28]
	/	/	0	NA	NA	111	HRM and Sanger sequencing	/	Fish et al., 2016 [29]
*FLNC*	c.2971C>T	p.Arg991*	1	2	1	120	WES	P	Hall et al., 2019 [30]
	c.7252-1G>A	/	1	0	1			LP	
	c.4718T>A	p.Leu1573*	1	2	2			P	
	c.1444C>T	p.Arg482*	1	4	0			P	
	c.180_191del	p.Gln61_Leu64del	1	1	0			VUS	
	c.6779A>G	p.Lys2260Arg	1	0	1			VUS	
	c.6173A>G	p.Gln2058Arg	1	0	0			VUS	
	c.2141T>C	p.Ile714Thr	1	0	0			VUS	
	c.5644A>G	p.Ile1882Val	1	0	0			VUS	
	c.249C>G	p.Tyr83*	1	0	1	219	NGS panel	LP	Ortiz-Genga et al., 2016 [10]
	c.4127+1del	/	1	5	0			LP	
	c.1714C>T	p.Gln572*	1	0	0			LP	
	c.5398G>T	p.Gly1800*	1	3	2			LP	
	c.4580+1G>T	/	1	0	0			LP	
	c.241del	p.Arg81Alafs*15	1	1	1			LP	
	c.6565G>T	p.Glu2189*	1	1	0	156	NGS panel + WGS	LP	Brun et al., 2020 [31]
	c.8107del	p.Asp2703Thrfs*69	1	0	0			LP	
	c.3791-1G>C	/	3	5	0	3	NGS panel + WGS	P	Oz et al., 2020 [32]
*CDH2*	c.1219G>A	p.Asp407Asn	1	3	0	4	WES + Sanger sequencing	P	Turkowski et al., 2017 [11]
	c.686A>C	p.Gln229Pro	1	5	0	74	WES + Sanger sequencing	LP	Mayosi et al., 2017 [12]
	c.614C>T	p.Pro205Leu	1	2	NA	500	NA	LP	Ghidoni et al., 2021 [33]
	c.781A>G	p.Asn261Asp	1	2	NA			LP	
	c.1973dup	p.Asn658Lysfs*3	1	NA	NA			P	
	c.2035C>G	p.Pro479Ala	1	1	0			LP	
	c.2137G>C	p.Asp713His	1	NA	NA			B	
	c.2167G>A	p.Gly723Ser	1	NA	NA			VUS	
*TJP1*	c.2006A>G	p.Tyr669Cys	1	3	4	40	WES + Sanger sequencing	VUS	De Bortoli et al., 2018 [13]
	c.793C>T	p.Arg265Trp	1	2	0			VUS	

TMEM43: transmembrane protein 43; DES: desmin; FLNC: filamin C; CDH2: cadherin-2; HGVS: Human Genome Variation Society; WES: whole exome sequencing; DGGE: denaturing gradient gel electrophoresis. VUS: variant of uncertain significance; LP: likely pathogenic; P: pathogenic; B: benign.

**Table 3:** Burden test of non-synonymous rare genetic variants in ACM population.

Gene	Variants	ACM Alleles	Ctrl Alleles	OR (95% CI)	*p*-Value
*TMEM43*	Non-synonymous	28	436	3.79 (2.58 to 5.57)	<0.0001
	No variants	1746	102,949		
*DES*	Non-synonymous	27	249	10.31 (6.93 to 15.31)	<0.0001
	No variants	972	92,432		
*FLNC*	Non-synonymous	42 (31 radical)	1927 (18 radical)	1.354 * (107 radical) (0.99 to 1.85) (59.75 to 191.7 radical)	0.0564 (<0.0001 radical)
	No variants	1636	101,669		
*CDH2*	Non-synonymous	10	501	1.138 (0.61 to 2.13)	0.6091
	No variants	1796	102,379		
*TJP1*	Non-synonymous	2	1093	0.258 (0.06 to 1.04)	0.05
	No variants	720	101,509		
*PLN*	Non-synonymous	31	20	117.8 (66.95 to 207.1)	<0.0001
	No variants	1340	101,808		

* Significant differences were annotated between FLNC missense and radical variants. ACM allele count is calculated by combining cases in the literature and the Padua cohort. GnomAD database was considered as control population. Fischer’s exact test and odd ratio calculation were performed for contingency analysis. Ctrls: control cases from gnomAD population; OR: Odd ratio.

## Data Availability

The data that support the findings of this study are available on request from the corresponding author.

## Supplementary Materials

The following supporting information can be downloaded at: https://www.mdpi.com/article/10.3390/ijms25116267/s1. Refs. [55,56,57,58,59,60] are cited in Supplementary Materials file.
