# Supplementary material for: A Comprehensive Analysis of Non-Desmosomal Rare Genetic Variants in Arrhythmogenic Cardiomyopathy: Integrating in Padua Cohort Literature-Derived Data
Source: Int J Mol Sci. 2024 Jun 6;25(11):6267. doi: 10.3390/ijms25116267 (PMC11173278; doi:10.3390/ijms25116267)
Supplement: Supplementary file 1 [file ijms-25-06267-s001.zip › ijms-3028414-supplementary.pdf]

Supplementary Materials and Methods

Genetic screening

DNA extraction from whole blood was performed on MagNA PURE Compact Nucleic acid (Roche, Germany) according to the user manual. DNA concentration, purity and integrity of each sample was measured using Qubit fluorometer (Thermo Fisher Scientific, USA), NanoDrop One spectrophotometer (ThermoFisher Scientific, USA) and TapeStation 4200 (Agilent, USA) respectively.

Targeted Next Generation Sequencing (NGS) was performed in all ACM patients by using TruSight Cardio Sequencing Kit (Illumina, USA), which covers 575 Kb of genomic DNA, the exon sequences of 174 genes associated with 17 inherited cardiac conditions: cardiomyopathies, arrhythmias, aortopathies, and channelopathies, among which *TMEM43* (NC_000003.11, NM_024334.2), *DES* (NC_000002.11, NM_001927.3) and *PLN* (NC_000006.11, NM_002667.4). Library preparation was performed according to manufacturer's instructions and sequenced on MiSeq (Illumina, USA).

The coding region and exon flanking regions of *FLNC* (NC_000007.13, NM_001458), *CDH2* (NC_000018.9, NM_001792.4) and *TJP1* (NC_000015.9, NM_003257.4) genes in all 320 ACM patients were analyzed by a custom panel (Illumina, USA). Variants that passed internal quality and frequency filters was confirmed by direct Sanger sequencing on AB3500Dx Genetic Analyzer (ThermoFisher Scientific, USA) as previously reported (1).

Variant calling and prioritization

Demultiplexing of sequence data was performed using Miseq Control software, sequences were aligned to the GRCh37/hg19 reference genome using Burrows-Wheeler Alignment tool (BWA v0.7.10) and GATKv3.2-2 was used for variant calling process. Information regarding frequency in different populations (1,000 Genomes Project, Exome Variant Server, Exome Aggregation Consortium, gnomAD database) was considered.

The allele frequency threshold to consider a variant clinically relevant was <0.01% since the estimated prevalence of the disease ranges about 1:2000 - 1:5000. The threshold was further adapted to the frequency of the most recurrent ACM variant (*PKP2*: c.2146-1G>C) and the algorithm proposed by Whiffin et al (2) which estimates the expected frequency of ACM variants at 6.7×10^{-5} ($4.1–9.2 \times 10^{-5}$).

Variants were described based on the current Human Genome Variation Society mutation nomenclature guidelines using the accession number of the longest transcript and the interpretation of these variants were classified according to the current ACMG recommendations (3, 4). Several algorithms were interrogated to assess variant pathogenicity: MutationTaster, SIFT, BayesDel, DANN, DEOGEN2, EIGEN, FATHMM, LIST-S2, LRT, M-CAP, MVP, MutPred, MetaRNN, Mutation Assessor, REVEL.

Splicing site assessment

Frozen right-ventricle (RV) myocardium was available from two ACM patients who underwent cardiac transplantation, one of them carries a splicing site variant in *CDH2*. Myocardial tissue was collected and immediately flash frozen and conserved at -80°C until used. Samples were homogenized using 5-mm steel beads in a TissueLyser instrument (Qiagen, Germany; 2 cycles of 2 minutes at 25 mHz). The homogenized samples were then incubated with 20 µl of Proteinase K (20 mg/mL; Roche Diagnostics GmbH, Mannheim, Germany) for 10 min at 56°C. Subsequently 200 µl of chloroform was added in each homogenized sample which was then centrifuged at 12000 rcf for 15 min at 4°C. Nucleic acids were purified using Spin column of the RNeasy mini kit (Qiagen, Germany) with a 20-minute on-column DNase I digestion (Qiagen, Germany). RNA concentration was determined by using Qubit fluorometer (Life Technologies, USA).

Subsequent reverse transcription of RNA samples was performed with SuperScript II (Thermo Fisher Scientific, USA) according to user manual. Primers were design with Primer-Blast, specifically within exon 14 and exon 15 regions of *CDH2* gene to exclusively amplified cDNA (Figure S4). Quantitative PCR assays were conducted in triplicate in a Light Cycler 480 II instrument (Roche, USA) and *GAPDH* gene was used as housekeeping gene due to its ubiquitous expressivity. Relative quantification was used to compare *CDH2* expression.

Evolutionary conservation

Selecton server was used to investigate evolutionary conservation of aminoacidic sequence of TMEM43, DES, PLN, FLNC, CDH2, and TJP1. Coding sequences were collected from Ensembl (5) from at least 10 different species and site-specific rate of non-synonymous substitutions (Ka) to the rate of synonymous substitutions (Ks) were computed at each site in orthologous sequences (Ka/Ks) as previously described (6). Aminoacidic sequence and Ka/Ks ratio were plotted, representing missense variants found in our cohort and in literature. A Ka/Ks ratio of 1 indicates neutral or no selection pressure, when ratio is lower than 1 indicates that amino acid residues are under negative selection pressure and when it is higher than 1, means positive selection. As such, when a missense variant involves an amino acid under positive selection, its change should not alter protein function/structure. On the other hand, when this occurs in a residue under negative selection pressure and as consequence, more conservative site, this should alter protein conformation and function.

Supplementary Results

TMEM43 cascade genetic screening

Transmembrane protein 43 was linked to ACM for the first time by Merner and co-workers in 2008 (7), describing the founder mutation Ser358Leu (c.1073C>T) in 15 unrelated ACM patients from Newfoundland in Canada. Since then, this has been the only pathogenic variant reported in ACM with a total of 22 patients/families out of 553 investigated index cases (Table 1).

Cascade genetic screening in family A (Figure S1, #4, II-1) was not informative. In family B, the 45-yr old c.1073C>T carrier (Figure S1, #2, III-2, female) had also another VUS at the position c.2651A>T of *Vinculin* (*VCL* [MIM: 193065]) gene. Her sister affected by dilated cardiomyopathy (DCM), carried only this *VCL* VUS but not the founder *TMEM43* variant. In family C, the 30-yr old proband (Figure S1, #3, III-1, male) was the only symptomatic carrier, both his mother and grandfather did not exhibit signs of the disease while his grandmother was affected by DCM and resulted to be *TMEM43* negative. In family D, we identified the missense c.349A>G (exon 4, Arg117Gly) variant in a 22-yr old patient with definite TFC but without family history of ACM or SCD (Figure S1 – family D, #1, II-1, male). This well-conserved rare variant (Figure 1) is neither reported in the literature nor found in gnomAD population database, and numerous pathogenicity algorithms predicted the variant as damaging/disease-causing (Table 1). Based on current ACMG rules this variant was classified as VUS increasing background genetic noise.

DES cascade genetic screening

In family A (Figure S2), the missense variant c.322G>A carrier was a 35-yr old male (#8, II-2) who died suddenly with an autoptic diagnosis of ACM with ALVC phenotype. This rare missense variant was previously reported in another patient affected by DCM, even though immunofluorescence assay on patient specimens did not reveal DES cytoskeletal network pattern differences compared to controls (8). As such, conflicting interpretation of pathogenicity is attributed in ClinVar database albeit 21 *in silico* pathogenicity tools predict this variant as damaging/disease causing. The pathogenicity of this variant was further supported by cascade family screening showing co-segregation (Figure S2), since his 15-yr old son (III-2) and 42-yr old brother (II-1) exhibited late-gadolinium enhancement in the left ventricle (LV) upon contrast-enhanced cardiac magnetic resonance (CE-CMR).

The missense variant c.346A>G carrier is a 17-yr old male (#9) diagnosed as ACM with ALVC pattern who underwent cardiac transplantation for refractory heart failure (Figure 2). This well-conserved rare variant (Figure 1) encoding for p.Asp116Asn, was previously described in a patient affected by end-stage ACM with heart failure (9) and it was predicted as damaging/disease-causing by numerous pathogenicity algorithms leading to its classification as LP.

In family B (Figure S2), the missense variant c.1009G>A carrier was a 29-yr old male (#11, II-2) who had a sex-triggered SCD but no family history for cardiac diseases or SCD. Histologic findings of heart specimen supported an ACM diagnosis with ALVC pattern. This rare missense variant identified in *DES* exon 5 is leading to p.Ala337Thr replacement into the highly conserved ROD 2B protein domain which is important for correct filament assembly and dimer-dimer interactions of the mature DES filament (10, 11). Even though cascade screening was not informative, several algorithms supported its pathogenicity allowing its classification as LP. Interestingly, a different aminoacidic change at the same position has been recently reported (p.Ala337Pro) in a family affected by left-ventricular non-compaction cardiomyopathy, as to reflect the huge heterogeneity among *DES*-carriers (12).

The missense variant c.1064G>A carrier (#10), is a 34 yr-old male with definite TFC. This rare variant, located in exon 6 of *DES* (ROD 2B domain), is causing the p.Arg355Pro replacement within a highly conserved position (Figure 1). It has been previously described in a patient with muscle weakness and conduction abnormalities. Ultrastructural analysis showed granulo-filamentous structures surrounding intercalated discs irrespective of the contracted or extended state of cardiomyocytes (13). As such this variant was classified as LP.

An *in-frame* deletion of three aminoacids p.Val126_Leu129del (c.377_388delTGCGCTTCCTGG) was found in a 35-year old woman with SCD (#13). Autoptic findings showed ALVC. Variant localizes in exon 1 within the ROD-1A domain. According to ACMG rules was classified as LP, as it is absent from gnomAD and not reported in literature.

The missense variant c.250G>A carrier (#7) is a 39-yr old female, displaying an ACM pattern with predominant involvement of the LV, leading to the amino acidic replacement p.Gly84Ser. This variant was classified as VUS since no family members were available for co-segregation analysis.

FLNC cascade genetic screening

Proband #26, is a 63 yr old male (Figure S3-Family A, III-8) who had ACM diagnosis at the age of 39 yr-old after an arrhythmic storm. He is carrying the c.5398+1 G>T *FLNC* variant in exon 32 which is predicted to alter the canonical donor splicing site of intron 32 (HSF CV variation -33.93%, TrapScore 0.938) and to activate a cryptic donor site (CV variation 67.41%). Loss-of-function (LOF) is a known mechanism of ACM, and this gene displayed a LOF Z-Score of about 7.96. This specific variant is located on the Ig-like repeat 11 (R11) of ROD1 domain which forms with Ig-like repeat 12 (R12) creating one of the most compacted structures in *FLNC* (14). This domain pair (R11-R12) together with R3-R5 and R14-R15 can be unfolded by mechanical forces to expose cryptic binding sites (mechanotransduction). As such, this radical variant was classified as LP. Cascade genetic screening within the family showed that *FLNC* variant co-segregates with phenotype in other 7 family members.

Proband #16, a 59 yr old male (Figure S3-Family B, II-2) who had ACM diagnosis at the age of 40, is carrying the missense c.1373 C>G variant in exon 8 encoding for the p.Pro458Arg substitution at the *FLNC* protein. This protein defect is localized in the Ig-like repeat 2 (R2) of ROD1 domain without a specific known function. Only 11 of the 21 *in silico* predictive algorithms interrogated classified this missense variant as P, thus, PP3 criterion could not be ascribed, and the variant was classified as VUS due to its absence in gnomAD. All family carriers are asymptomatic without any clinical signs of ACM.

Proband #22, a basketball male player who died suddenly at rest at the age of 17 (Figure S3-Family C, II-2), carried the nonsense c.5926C>T *FLNC* variant in exon 36 encoding for p.Gln1976Ter. This radical variant is localized on Ig-like repeat 16 (R16) of the ROD2 protein domain and it was classified as P. Detailed autoptic, and histologic examination revealed ACM with left-dominant pattern. No family history of the disease or Sudden cardiac death (SCD) were reported.

Proband #24, 59 yr old female (Figure S3-Family D, II-2) who underwent genetic screening due to SCD of her sister at the age of 56 (II-1), carried the c.1623_1624insT *FLNC* variant in exon 10 which is leading to a premature stop codon after 21 aminoacids of the protein (p.Pro542SerfsTer21). Clinical evaluation of the proband showed T wave inversion in V1-V6 leads in ECG but no significant functional and/or tissue alterations although autopsy findings on the sister's heart were compatible with ACM. This radical variant is located in the Ig-like repeat 3 (R3) of the ROD1 domain, which linked to R5 is hiding cryptic binding sites for filamin-partner complexes as previously acknowledged for the splicing site found in family A. This variant was classified as LP.

Proband #23, a 44 yr old male (Figure S3-Family E, III-1) with family history of SCD, carried the c.3781 G>T variant in exon 21. Based on ACMG criteria this variant was classified as LP. This nonsense variant is leading to a premature stop codon p.Glu1261Ter, located in Ig-like repeat 18 of the ROD2 domain, which is essential for FLNC dimerization and Z-disc interaction. As such this variant probably invokes haploinsufficiency via nonsense-mediated decay, leading to sarcomere disarray and weakened cell-cell adhesion with subsequent impaired mechanotransduction. The proband's father (II-2) died at the age of 27 (no other data) and his cousin carries FLNC variant and was found to be affected by ACM with left-dominant pattern. His asymptomatic mother was genotype negative.

CDH2 cascade genetic screening

In *CDH2*, we found the intronic c.2349+4A>G variant affecting the donor splicing site of the protein (HSF CV variation -10.93%, TrapScore: 0.973) in a 62 yr old woman with definite ACM diagnosis (proband #38). This splicing site variant is not reported in gnomAD database and its assessment by qPCR of exon14-15 demonstrated that this radical variant does not lead to mRNA decay since similar levels were observed between patient's and healthy control samples (Figure S4). As such, it was classified as VUS /likely benign. Another rare variant of *CDH2* c.*92T>A was identified in a 37 yr old male exhibiting ALVC pattern (proband #39). Since this non-coding nucleotide variant was identified at the 3'UTR of the protein and it was absent in gnomAD, it was classified as VUS.

References

1. Pilichou K, Lazzarini E, Rigato I, Celeghin R, De Bortoli M, Perazzolo Marra M, et al. Large Genomic Rearrangements of Desmosomal Genes in Italian Arrhythmogenic Cardiomyopathy Patients. Circ Arrhythm Electrophysiol. 2017;10(10).
2. Whiffin N, Minikel E, Walsh R, O'Donnell-Luria AH, Karczewski K, Ing AY, et al. Using high-resolution variant frequencies to empower clinical genome interpretation. Genetics in medicine : official journal of the American College of Medical Genetics. 2017;19(10):1151-8.
3. Hershberger RE, Givertz MM, Ho CY, Judge DP, Kantor PF, McBride KL, et al. Genetic evaluation of cardiomyopathy: a clinical practice resource of the American College of Medical Genetics and Genomics (ACMG). Genetics in medicine : official journal of the American College of Medical Genetics. 2018;20(9):899-909.
4. Richards S, Aziz N, Bale S, Bick D, Das S, Gastier-Foster J, et al. Standards and guidelines for the interpretation of sequence variants: a joint consensus recommendation of the American College of Medical Genetics and Genomics and the Association for Molecular Pathology. Genetics in medicine : official journal of the American College of Medical Genetics. 2015;17(5):405-24.
5. Howe KL, Achuthan P, Allen J, Allen J, Alvarez-Jarreta J, Amode MR, et al. Ensembl 2021. Nucleic acids research. 2021;49(D1):D884-D91.
6. Cason M, Celeghin R, Marinas MB, Beffagna G, Della Barbera M, Rizzo S, et al. Novel pathogenic role for galectin-3 in early disease stages of arrhythmogenic cardiomyopathy. Heart rhythm. 2021.
7. Merner ND, Hodgkinson KA, Haywood AF, Connors S, French VM, Drenckhahn JD, et al. Arrhythmogenic right ventricular cardiomyopathy type 5 is a fully penetrant, lethal arrhythmic disorder caused by a missense mutation in the TMEM43 gene. American journal of human genetics. 2008;82(4):809-21.
8. Taylor MR, Slavov D, Ku L, Di Lenarda A, Sinagra G, Carniel E, et al. Prevalence of desmin mutations in dilated cardiomyopathy. Circulation. 2007;115(10):1244-51.
9. Klauke B, Kossmann S, Gaertner A, Brand K, Stork I, Brodehl A, et al. De novo desmin-mutation N116S is associated with arrhythmogenic right ventricular cardiomyopathy. Human molecular genetics. 2010;19(23):4595-607.
10. Herrmann H, Aebi U. Intermediate Filaments: Structure and Assembly. Cold Spring Harbor perspectives in biology. 2016;8(11).
11. Strelkov SV, Herrmann H, Aebi U. Molecular architecture of intermediate filaments. BioEssays : news and reviews in molecular, cellular and developmental biology. 2003;25(3):243-51.
12. Kulikova O, Brodehl A, Kiseleva A, Myasnikov R, Meshkov A, Stanasiuk C, et al. The Desmin (DES) Mutation p.A337P Is Associated with Left-Ventricular Non-Compaction Cardiomyopathy. Genes. 2021;12(1).
13. Fidzianska A, Kotowicz J, Sadowska M, Goudeau B, Walczak E, Vicart P, et al. A novel desmin R355P mutation causes cardiac and skeletal myopathy. Neuromuscular disorders : NMD. 2005;15(8):525-31.
14. Nakamura F, Osborn TM, Hartemink CA, Hartwig JH, Stossel TP. Structural basis of filamin A functions. J Cell Biol. 2007;179(5):1011-25.
15. Christensen AH, Andersen CB, Tybjaerg-Hansen A, Haunso S, Svendsen JH. Mutation analysis and evaluation of the cardiac localization of TMEM43 in arrhythmogenic right ventricular cardiomyopathy. Clinical genetics. 2011;80(3):256-64.
16. Baskin B, Skinner JR, Sanatani S, Terespolsky D, Krahn AD, Ray PN, et al. TMEM43 mutations associated with arrhythmogenic right ventricular cardiomyopathy in non-Newfoundland populations. Human genetics. 2013;132(11):1245-52.
17. Haywood AF, Merner ND, Hodgkinson KA, Houston J, Syrris P, Booth V, et al. Recurrent missense mutations in TMEM43 (ARVD5) due to founder effects cause arrhythmogenic cardiomyopathies in the UK and Canada. European heart journal. 2013;34(13):1002-11.

18. van Tintelen JP, Van Gelder IC, Asimaki A, Suurmeijer AJ, Wiesfeld AC, Jongbloed JD, et al. Severe cardiac phenotype with right ventricular predominance in a large cohort of patients with a single missense mutation in the DES gene. Heart rhythm. 2009;6(11):1574-83.
19. Otten E, Asimaki A, Maass A, van Langen IM, van der Wal A, de Jonge N, et al. Desmin mutations as a cause of right ventricular heart failure affect the intercalated disks. Heart rhythm. 2010;7(8):1058-64.
20. Hedberg C, Melberg A, Kuhl A, Jenne D, Oldfors A. Autosomal dominant myofibrillar myopathy with arrhythmogenic right ventricular cardiomyopathy 7 is caused by a DES mutation. European journal of human genetics : EJHG. 2012;20(9):984-5.
21. Lorenzon A, Beffagna G, Bauce B, De Bortoli M, Li Mura IE, Calore M, et al. Desmin mutations and arrhythmogenic right ventricular cardiomyopathy. The American journal of cardiology. 2013;111(3):400-5.
22. Ripoll-Vera T, Zorio E, Gamez JM, Molina P, Govea N, Cremer D. Phenotypic Patterns of Cardiomyopathy Caused by Mutations in the Desmin Gene. A Clinical and Genetic Study in Two Inherited Heart Disease Units. Revista espanola de cardiologia. 2015;68(11):1027-9.
23. Bermudez-Jimenez FJ, Carriel V, Brodehl A, Alaminos M, Campos A, Schirmer I, et al. Novel Desmin Mutation p.Glu401Asp Impairs Filament Formation, Disrupts Cell Membrane Integrity, and Causes Severe Arrhythmogenic Left Ventricular Cardiomyopathy/Dysplasia. Circulation. 2018;137(15):1595-610.
24. Protonotarios A, Brodehl A, Asimaki A, Jager J, Quinn E, Stanasiuk C, et al. The novel desmin variant p.Leu115Ile is associated with a unique form of biventricular Arrhythmogenic Cardiomyopathy. Can J Cardiol. 2020.
25. van der Zwaag PA, van Rijsingen IA, Asimaki A, Jongbloed JD, van Veldhuisen DJ, Wiesfeld AC, et al. Phospholamban R14del mutation in patients diagnosed with dilated cardiomyopathy or arrhythmogenic right ventricular cardiomyopathy: evidence supporting the concept of arrhythmogenic cardiomyopathy. Eur J Heart Fail. 2012;14(11):1199-207.
26. Groeneweg JA, van der Zwaag PA, Olde Nordkamp LR, Bikker H, Jongbloed JD, Jongbloed R, et al. Arrhythmogenic right ventricular dysplasia/cardiomyopathy according to revised 2010 task force criteria with inclusion of non-desmosomal phospholamban mutation carriers. The American journal of cardiology. 2013;112(8):1197-206.
27. Fish M, Shaboodien G, Kraus S, Sliwa K, Seidman CE, Burke MA, et al. Mutation analysis of the phospholamban gene in 315 South Africans with dilated, hypertrophic, peripartum and arrhythmogenic right ventricular cardiomyopathies. Scientific reports. 2016;6:22235.
28. Hall CL, Akhtar MM, Sabater-Molina M, Futema M, Asimaki A, Protonotarios A, et al. Filamin C variants are associated with a distinctive clinical and immunohistochemical arrhythmogenic cardiomyopathy phenotype. International journal of cardiology. 2019.
29. Ortiz-Genga MF, Cuenca S, Dal Ferro M, Zorio E, Salgado-Aranda R, Climent V, et al. Truncating FLNC Mutations Are Associated With High-Risk Dilated and Arrhythmogenic Cardiomyopathies. Journal of the American College of Cardiology. 2016;68(22):2440-51.
30. Brun F, Gigli M, Graw SL, Judge DP, Merlo M, Murray B, et al. FLNC truncations cause arrhythmogenic right ventricular cardiomyopathy. Journal of medical genetics. 2020;57(4):254-7.
31. Oz S, Yonath H, Visochyk L, Ofek E, Landa N, Reznik-Wolf H, et al. Reduction in Filamin C transcript is associated with arrhythmogenic cardiomyopathy in Ashkenazi Jews. International journal of cardiology. 2020;317:133-8.
32. Turkowski KL, Tester DJ, Bos JM, Haugaa KH, Ackerman MJ. Whole exome sequencing with genomic triangulation implicates CDH2-encoded N-cadherin as a novel pathogenic substrate for arrhythmogenic cardiomyopathy. Congenit Heart Dis. 2017;12(2):226-35.
33. Mayosi BM, Fish M, Shaboodien G, Mastantuono E, Kraus S, Wieland T, et al. Identification of Cadherin 2 (CDH2) Mutations in Arrhythmogenic Right Ventricular Cardiomyopathy. Circulation Cardiovascular genetics. 2017;10(2).

34. Ghidoni A, Elliott PM, Syrris P, Calkins H, James CA, Judge DP, et al. Cadherin 2-Related Arrhythmogenic Cardiomyopathy: Prevalence and Clinical Features. Circulation Genomic and precision medicine. 2021.
35. De Bortoli M, Postma AV, Poloni G, Calore M, Minervini G, Mazzotti E, et al. Whole-Exome Sequencing Identifies Pathogenic Variants in TJP1 Gene Associated With Arrhythmogenic Cardiomyopathy. Circulation Genomic and precision medicine. 2018;11(10):e002123.

Figure S1 *TMEM43* pedigrees.

Four family trees showing *TMEM43* gene variant. Light blue squares and circles indicate affected males and females, respectively. White squares and circles indicate healthy males and females, respectively. The +/- sign indicates heterozygous *TMEM43* carrier; The * indicates heterozygous VCL carriers. The arrow indicates the proband; and the crossed-out square or circle indicates that the subject is dead. Brief clinical informations are reported, nsVT: non-sustained Ventricular Tachycardia; ECHO echocardiography; ECG: electrocardiogram; LV: Left Ventricle; LGE: Late Gadolinium Enhancement; LBBB: Left Ventricular Bundle Branch Block; EF: Ejection Fraction; TWI: T-wave inversion

Figure S2 *DES* pedigrees.

Two family trees showing *DES* gene variant segregation. Light blue squares and circles indicate affected males and females, respectively. White squares and circles indicate healthy males and females, respectively. The +/- sign indicates heterozygous *DES* carrier. The arrow indicates the proband; and the crossed-out square or circle indicates that the subject is dead. Brief clinical information are reported, ECHO echocardiography; LV: Left Ventricle; LGE: Late Gadolinium Enhancement.

Figure S3*: *FLNC pedigrees.

Five family trees showing *FLNC* gene variant segregation. Light blue squares and circles indicate affected males and females, respectively. White squares and circles indicate healthy males and females, respectively. The +/- sign indicates heterozygous *FLNC* carrier. The arrow indicates the proband; and the crossed-out square or circle indicates that the subject is dead. Brief clinical informations are reported. LV: Left Ventricle; BIV: biventricular; LGE: Late Gadolinium Enhancement; ECHO echocardiography; SCD: Sudden Cardiac Death; Tx: transplantation.

Figure S4: Splicing site analysis of genetic variant *CDH2* c.2349+4A>G.

Log_2Fold-change of *CDH2* expression in case and control. P-value calculated by Mann-Whitney test.

Figure S1

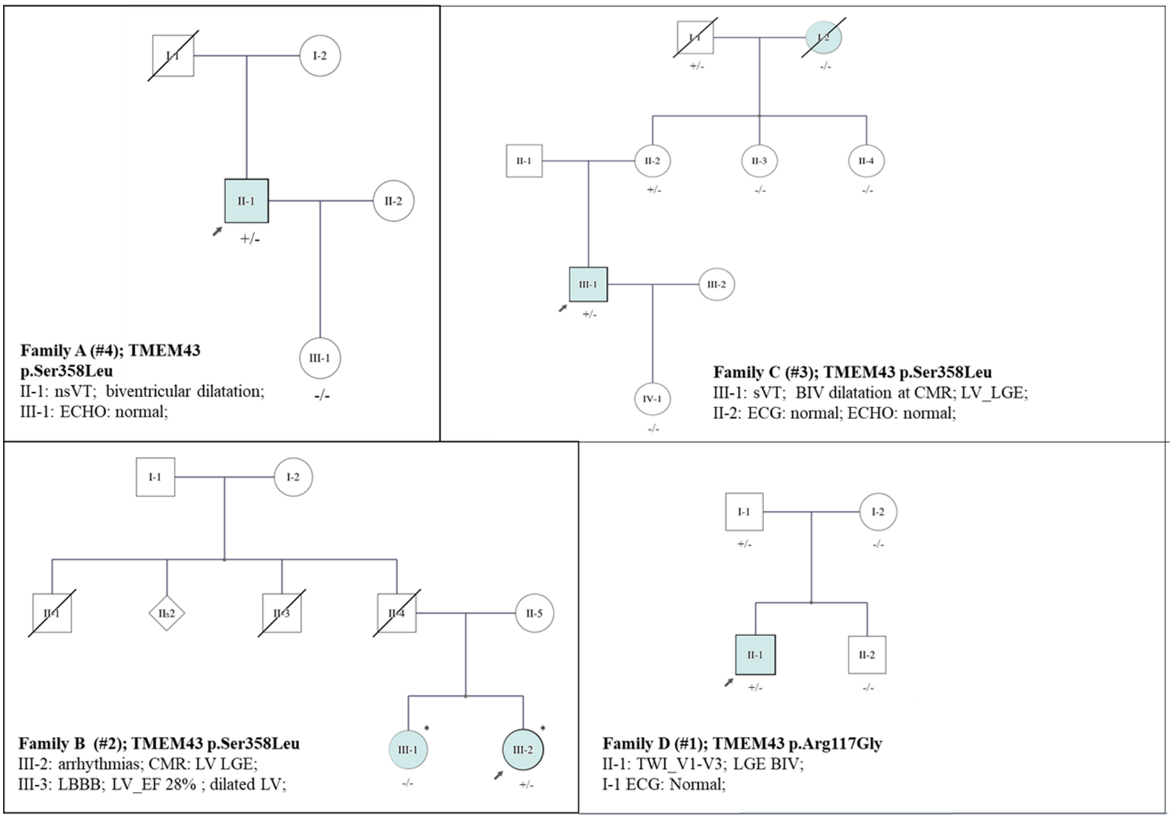
I-2
II-1
+/-
II-2
III-1
-/-
Family A (#4); TMEM43
p.Ser358Leu
II-1: nsVT; biventricular dilatation;
III-1: ECHO: normal;
+/-
-/-
II-1
II-2
II-3
II-4
+/-
-/-
-/-
III-1
III-2
+/-
IV-1
-/-
Family C (#3); TMEM43 p.Ser358Leu
III-1: sVT; BIV dilatation at CMR; LV_LGE;
II-2: ECG: normal; ECHO: normal;
I-1
I-2
II-5
III-1
III-2
-/-
+/-
Family B (#2); TMEM43 p.Ser358Leu
III-2: arrhythmias; CMR: LV LGE;
III-3: LBBB; LV_EF 28% ; dilated LV;
I-1
I-2
+/-
-/-
II-1
II-2
+/-
-/-
Family D (#1); TMEM43 p.Arg117Gly
II-1: TWI_V1-V3; LGE BIV;
I-1 ECG: Normal;

Figure S2

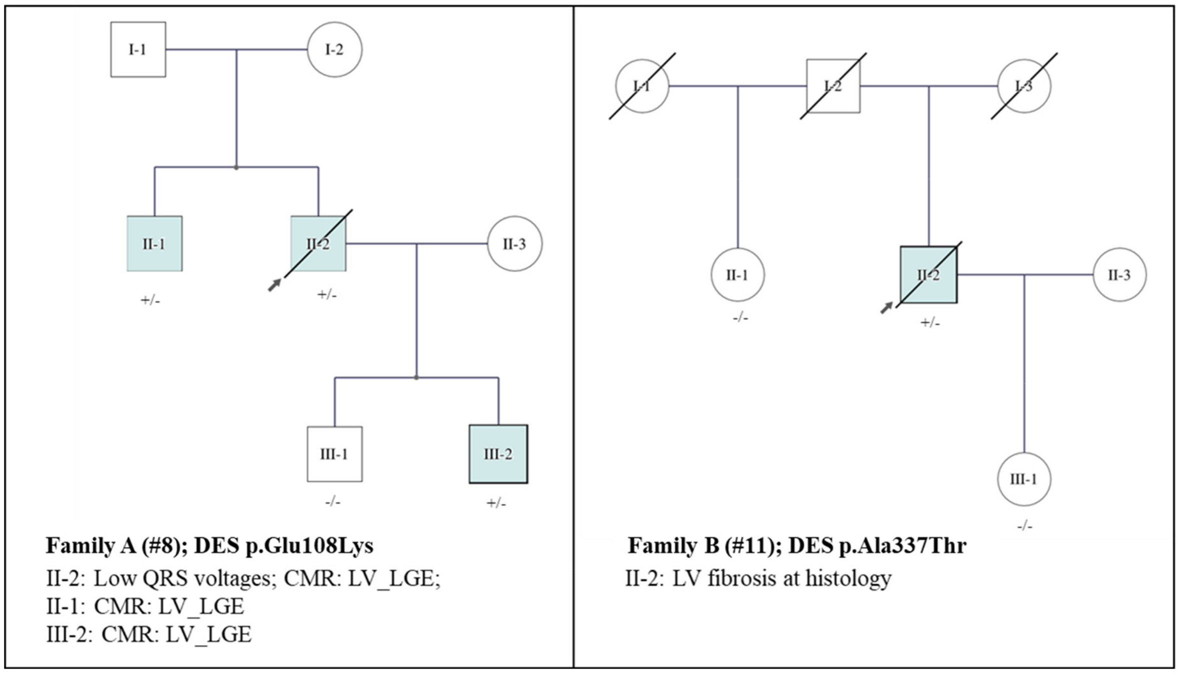

Family A (#8); DES p.Glu108Lys
II-2: Low QRS voltages; CMR: LV_LGE;
II-1: CMR: LV_LGE
III-2: CMR: LV_LGE

Family B (#11); DES p.Ala337Thr
II-2: LV fibrosis at histology

Figure S3

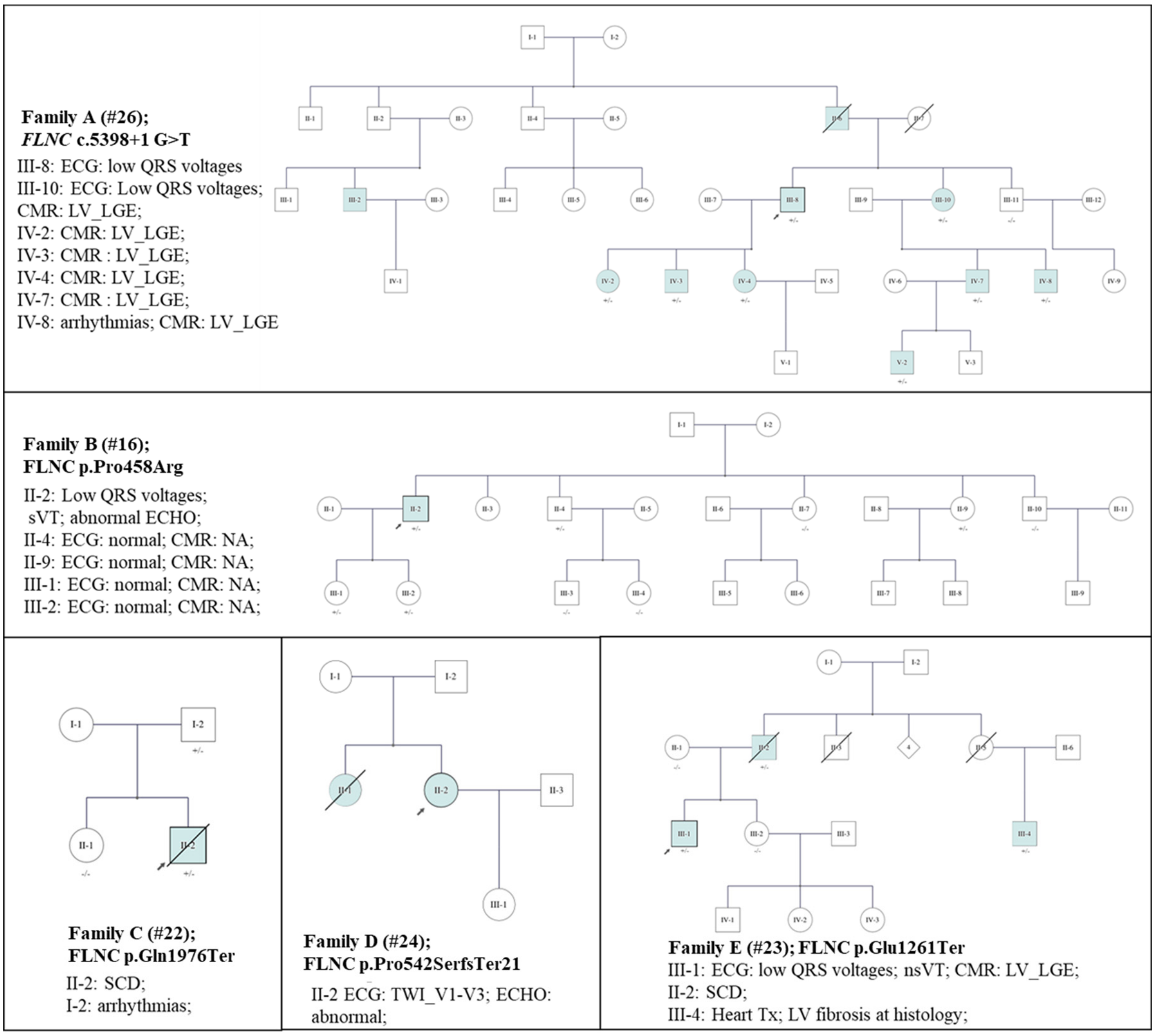
Family A (#26);
FLNC c.5398+1 G>T
III-8: ECG: low QRS voltages
III-10: ECG: Low QRS voltages; CMR: LV_LGE;
IV-2: CMR: LV_LGE;
IV-3: CMR : LV_LGE;
IV-4: CMR: LV_LGE;
IV-7: CMR : LV_LGE;
IV-8: arrhythmias; CMR: LV_LGE
Family B (#16);
FLNC p.Pro458Arg
II-2: Low QRS voltages;
sVT; abnormal ECHO;
II-4: ECG: normal; CMR: NA;
II-9: ECG: normal; CMR: NA;
III-1: ECG: normal; CMR: NA;
III-2: ECG: normal; CMR: NA;
I-1
I-2
II-1
II-2
Family C (#22);
FLNC p.Gln1976Ter
II-2: SCD;
I-2: arrhythmias;
I-1
I-2
II-1
II-2
II-3
III-1
Family D (#24);
FLNC p.Pro542SerfsTer21
II-2 ECG: TWI_V1-V3; ECHO: abnormal;
Family E (#23); FLNC p.Glu1261Ter
III-1: ECG: low QRS voltages; nsVT; CMR: LV_LGE;
II-2: SCD;
III-4: Heart Tx; LV fibrosis at histology;

Figure S4

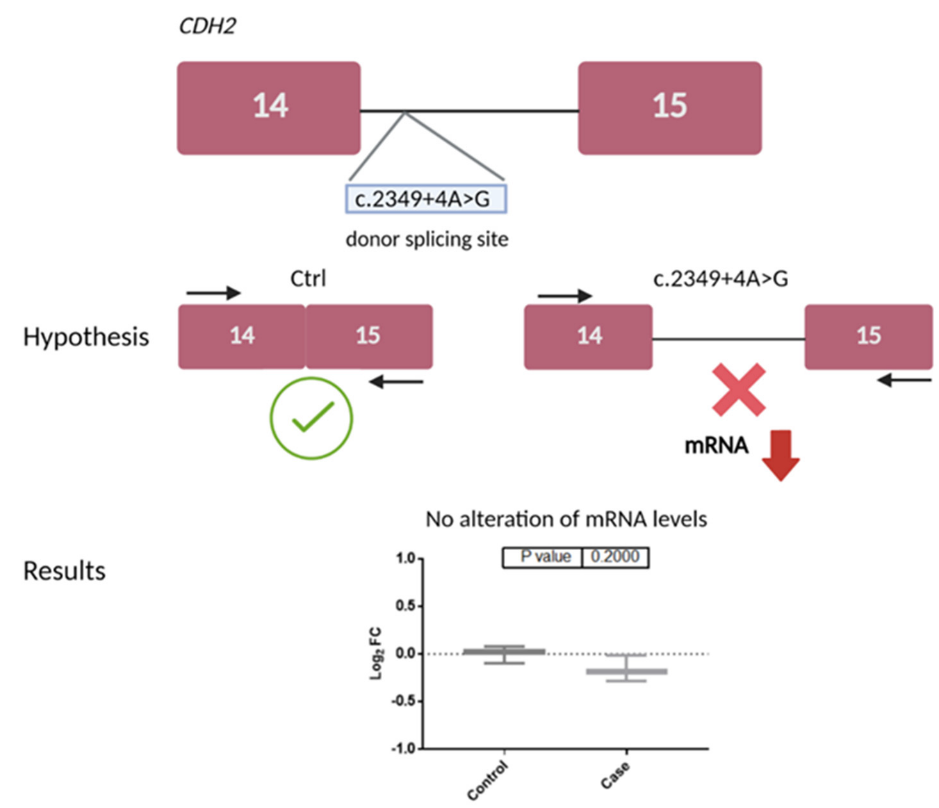
CDH2
14
15
c.2349+4A>G
donor splicing site
Ctrl
c.2349+4A>G
Hypothesis
14
15
14
15
mRNA
No alteration of mRNA levels
Results
P value
0.2000
1.0
0.5
0.0
-0.5
-1.0
Log2 FC
Control
Case
